# Meta-transcriptomic identification of hepatitis B virus in cerebrospinal fluid in patients with central nervous system disease

**DOI:** 10.1016/j.diagmicrobio.2019.114878

**Published:** 2019-12

**Authors:** John H.-O. Pettersson, Geraldine Piorkowski, Mayfong Mayxay, Sayaphet Rattanavong, Manivanh Vongsouvath, Viengmon Davong, Kristian Alfsnes, Vegard Eldholm, Xavier de Lamballerie, Edward C. Holmes, Paul N Newton, Audrey Dubot-Pérès

**Affiliations:** a Zoonosis Science Center, Department of Medical Biochemistry and Microbiology, Uppsala University, Uppsala, Sweden; b Marie Bashir Institute for Infectious Diseases and Biosecurity, Charles Perkins Centre, School of Life & Environmental Sciences and Sydney Medical School, The University of Sydney, Sydney, Australia; c Public Health Agency of Sweden, Nobels väg 18, SE-171 82, Solna, Sweden; d Infectious Diseases and Environmental Health, Norwegian Institute of Public Health, Lovisenberggata 8, Oslo 0456, Norway; e Unité des Virus Émergents (UVE: Aix-Marseille Univ – IRD 190 – Inserm 1207 – IHU Méditerranée Infection), Marseille, France; f Lao-Oxford-Mahosot Hospital-Wellcome Trust Research Unit (LOMWRU), Microbiology Laboratory, Mahosot Hospital, Vientiane, Lao PDR; g Institute of Research and Education Development (IRED), University of Health Sciences, Vientiane, Lao PDR; h Centre for Tropical Medicine and Global Health, Nuffield Department of Medicine, University of Oxford, Churchill Hospital, University of Oxford, Oxford, United Kingdom

**Keywords:** Hepatitis B virus, RNA sequencing, Meta-transcriptomics, Cerebrospinal fluid, Encephalitis

## Abstract

Determining the etiological basis of central nervous system (CNS) infections is inherently challenging, primarily due to the multi-etiological nature. Using RNA sequencing, we aimed to identify microbes present in cerebrospinal fluid (CSF) of two patients suffering CNS infection, previously diagnosed with *Cryptococcus* sp. and *Streptococcus pneumoniae* infection, respectively. After meta-transcriptomic analysis, and confirmation with real-time PCR, hepatitis B virus (HBV) was detected in the CSF of two patients diagnosed with CNS syndrome. Phylogenetic analysis of the partial HBV genomes from these patients showed that they belonged to genotypes B and C and clustered with other viruses of Asian origin. In countries with high levels of HBV endemicity, the virus is likely to be found in patients diagnosed with CNS infections, although whether it contributes to symptoms and pathology, or is simply a coincidental infection, is unknown and merits further investigation.

## 1 Background

Central nervous system (CNS) infections are responsible for potentially severe clinical presentations and are an important public health issue in South-east Asia due to their frequency, fatality rate, and frequency of sequelae (Dubot-Pérès et al. n.d.). The syndrome is complicated by the fact that various etiological agents are responsible for overlapping clinical presentations and co-infections are probably frequent, potentially including both agents that contribute to the severity of the disease as well as ‘passive’ co-infecting microbes whose the role in the disease is poorly understood. This makes accurate diagnosis inherently challenging (Glaser et al., 2006, Granerod et al., 2010, Dittrich et al., 2015). Importantly, over 50% of patients with suspicion of CNS infection remain undiagnosed following standard screening (Tan et al., 2014, Dittrich et al., 2015).

For those reasons, this syndrome also lends itself to meta-transcriptomic analysis, following RNA-seq (Wang et al., 2009, Ozsolak and Milos, 2011). This analysis allows for the identification and characterization of all the transcripts present in a sample (*i.e.* ‘meta-transcriptomics’) in an unbiased manner (Brown et al. 2018), including the identification of divergent and novel pathogens not revealed by standard molecular tools (Barzon et al., 2013, Houldcroft et al., 2017). Hence, meta-transcriptomics could provide a powerful way to further investigate the etiological agents associated with CNS infections.

Here we report two cases of CNS infection in which HBV co-infection was identified in the cerebrospinal fluid (CSF).

## 2 Objectives

The aim of our study was to assess the use of meta-transcriptomics for the unbiased detection of micro-organisms in two Lao patients presenting with suspected CNS infection.

## 3 Study design

### 3.1 Patient inclusion and sampling

Patients admitted to Mahosot Hospital, Vientiane, Laos, with suspected CNS infection, on the basis of altered consciousness or neurological findings, were recruited if a diagnostic lumbar puncture was indicated, without contraindications, and if they provided written informed consent. Eight milliliters of CSF were collected for adults (>15 year-old), tested for a broad range of biochemical and diagnostic assays (Dubot-Pérès et al. n.d.) and stored at −80 °C.

### 3.2 RNA extraction and sequencing

Total RNA was extracted from 50 μL of stored CSF using the *mir*Vana PARIS kit (ThermoFisher Scientific) following the manufacturer's instructions (elution volume: 100 μL). RNA was cleaned up and concentrated using TURBO DNA-*free* kit (Invitrogen) followed by RNeasy MinElute Cleanup Kit (Qiagen), respectively, according to the manufacturer's instructions (elution volume: 14 μL).

Paired-end Illumina libraries were constructed using the SMARTer Stranded Total RNA-Seq Kit v2 (Takara) following the manufacturer's instructions. Briefly, RNA was converted to cDNA followed by ligation of adapters with unique barcodes, depletion of ribosomal cDNA, PCR amplification and library clean-up. The libraries were sequenced on an Illumina HiSeq 2500 instrument (SciLifeLab, Uppsala, Sweden). Human reads were removed with Bowtie2 (Langmead and Salzberg 2012).

### 3.3 Pathogen identification

Sequence libraries were processed using an in-house pipeline that combines quality trimming, *de novo* assembly, and similarity based searches using the NCBI protein (nr) and nucleotide (nt) databases (Shi et al., 2016a, Eden et al., 2017, Pettersson et al., 2017). Tentative hits were further evaluated with searches against the NCBI conserved domain database, NCBI blastx and blastn searches, and mapping against the sequence libraries. MetaPhlAn2 (Segata et al. 2012) was used for bacterial identification and profiling. Potential pathogen contigs that were similar or identical to vectors and/or laboratory strains were excluded. Potential pathogens were confirmed, from the same RNA extract, by a specific probe based real-time PCR, as previously described for HBV (Loeb et al. 2000).

### 3.4 HBV genome analysis

To identify the publicly available HBV genomes most similar to those identified here, the *de novo* assembled HBV contigs found using the pathogen discovery pipeline were submitted to NCBI BLASTn. Using Bowtie2 (Langmead and Salzberg 2012), the most similar complete HBV genomes (MG571355 for sample L1 and KX774503 for sample L2, respectively) were then used as a reference to assemble HBV consensus genomes from the two HBV positive samples.

The consensus sequences of samples L1 and L2 were aligned with a set of representative complete genome HBV sequences, retrieved from NCBI GenBank, using Mafft v.7 (Katoh and Standley 2013). A phylogenetic tree was inferred using the maximum likelihood approach in PhyML v.3 (Guindon et al. 2010), employing the GTR nucleotide substitution model with a gamma distribution of rate variation, SPR branch-swapping and 1000 bootstrap replicates.

Partial HBV genome sequences from both patients are available on GenBank (MK286460 and MK286461). All raw read data is available at the NCBI short read archive (BioProject number: PRJNA509693).

## 4 Results

### 4.1 Patient description

Patient L1 and L2 were 22 and 19 year-old males admitted to hospital in 2009 and 2008 with 4 and 3 days of fever, respectively, and presented with clinical meningoencephalitis according to WHO criteria (World Health Organisation 2003). Both presented with neck stiffness, headache and reduced Glasgow Coma Scale score (13/15 for patient L1 and 11/15 for patient L2) (Table 1). *Cryptococcus* sp. infection was diagnosed for patient L1 based on positive cryptococcal Antigen Lateral Flow Assay from CSF. *Streptococcus pneumoniae* was detected in patient L2 using PCR (Carvalho et al. 2007) and culture from CSF and blood. Patient L1 received ceftriaxone, amphotericin B and doxycycline treatment and patient L2 with ceftriaxone. Both patients were discharged alive after 12 days of hospitalization.

**Table 1:** Characteristics of the two patients reported in the study.

	Patient L1	Patient L2
Age, years	22	19
Gender	male	male
Admission date	24th May 2009	16th March 2008
**Signs/symptoms**		
Days of fever on admission	4	3
History of jaundice	yes	yes
Neck stiffness	yes	yes
Glasgow Coma Scale score	13/15	11/15
History of seizure	No	No
WHO clinical CNS presentation	Meningoencephalitis	Meningoencephalitis
**CSF parameter**		
Total white cell count (cells/mm3)	40	210
Neutrophils (%)	38	100
Lymphocytes (%)	62	0
Red cell count (cells/ mm3)	0	95
HBV real-time PCR♦ (Cq value)	27	23
**Blood parameter**		
HBs antigen serology	NA	positive
ALT (IU/L)	34	26
AST (IU/L)	99	144
HBV real-time PCR£ (Cq value)	NA	20£
**Outcome**		
Etiological diagnosis⁎	*Cryptococcus* sp.	*Streptococcus pneumoniae*
Duration of hospitalization (days)	12	12
Discharge status	alive	alive

All HBV real-time PCR were performed the same way: from 5 μL of extract using system previously described (Loeb et al. 2000), using SuperScript™ III One-Step RT-PCR System with Platinum™ Taq DNA Polymerase (ThermoFisher), following manufacturer's instruction, 400 nM of each primer and 160 nM of probe, in final volume of 25 μL. Thermal cycling used was as followed: 50 °C for 15 min, 95 °C for 2 min and 45 cycles of 95 °C for 15 sec and 60 °C for 45 sec.

All available HBV investigations are presented in the table.

⁎ Laboratory confirmed by direct test in CSF, cryptococcal Antigen Lateral Flow Assay for patient L1, and real-time PCR and culture for patient L2.

♦ HBV real-time PCR was repeated on a new aliquot of CSF after extraction using EZ1 Virus Mini Kit V2.0 (Qiagen), same Cq value was obtained for patient L2 and Cq value of 28 was obtained for patient L1.

£ Serum sample from patient L2 (not available for patient L1) was extracted using EZ1 Virus Mini Kit V2.0 (Qiagen) then submitted to HBV real-time PCR.

### 4.2 Pathogen identification

**Fig. 1:**
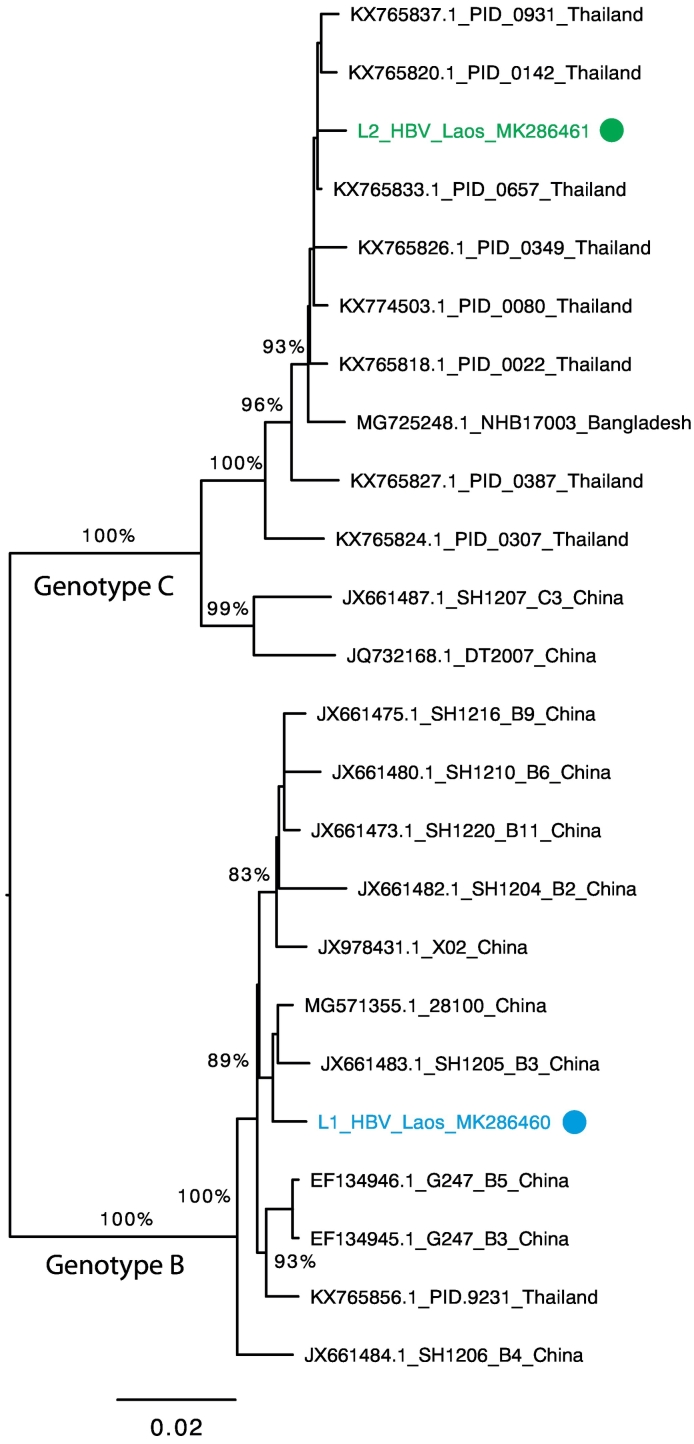
Phylogenetic tree of HBV from CSF from Lao patients L1 and L2 together with representative GenBank genome sequences of viral genotypes B and C that are commonplace in Asia. Numbers on branches indicate bootstrap support, and only branches with bootstrap support ≥80% are indicated. Branch lengths are scaled according to the number of nucleotide substitutions per site, and the tree is rooted between genotypes B and C.

Meta-transcriptomic analysis identified HBV in CSF of both patients L1 and L2. Reference based mapping produced two genetically distinct and near complete HBV genomes with a consensus length of 3215 nt (225 reads; mean coverage 7.8×) for sample L1 and a consensus length of 3158 nt (1098 reads; mean coverage 41×) for sample L2. Phylogenetic analysis positioned these two genomes in separate genotypes - HBV genotypes B (sample L1) and C (sample L2) – clustering with HBV sequences of Asian origin (Fig. 1). Importantly, HBV was identified in the CSF samples from both patients (L1 and L2) and confirmed by real-time PCR with Cq values of 27 and 23, respectively. Meta-transcriptomic data also detected diverse and abundant streptococcal bacteria, particularly *Streptococcus mitis, S. oralis,* and *S. pneumoniae,* in patient L2 CSF (data not shown), but could not definitively identify the *Cryptococcus* sp. infection in patient L1.

## 5 Discussion

Using an unbiased RNA-Seq approach we identified HBV co-infections in the CSF of two Lao patients with CNS infections conventionally diagnosed during admission with *Cryptococcus* sp. and *Streptococcus pneumoniae* infections. The HBV genotypes detected, B and C, are common in south-east Asia (Tian and Jia, 2016, Velkov et al., 2018). Given that HBV is endemic in many Asian countries (Schweitzer et al., 2015, World Health Organization, 2017) and the fact that HBV has been detected in CSF of patients, either with or without CNS-related symptoms (Spiegel et al., 1980, Inoue et al., 2008, Zhong et al., 2014, Ene et al., 2015, Pronier et al., 2018), it is likely that this virus will be present in some of the patients suffering from CNS syndrome as mixed infections (Phommasone et al. 2013).

HBV serology showed that patient L2 was positive for the hepatitis B surface antigen. HBV PCR performed on blood was also positive. No HBV investigation was available for patient L1, and blood samples were not available for additional testing. The HBV vaccine status for both patients is unknown. Patient L1 had no red blood cells in the CSF sample, whereas patient L2 had a red blood cell count of 95 red blood cells/μL in CSF, indicating that the HBV detected in the sample could have come from a traumatic tap. HBV may also have crossed the blood–brain barrier, and viral crossing of the blood–brain barrier has been shown in other hepatitis viruses, including hepatitis E virus and hepatitis C virus (Fletcher et al., 2012, Shi et al., 2016b).

Whether HBV, either as single- or co-infection, can induce or have a role in CNS disease remains uncertain. However Pronier et al. provided arguments in favor of HBV involvement in CNS disease (Pronier et al. 2018). The identification of credible etiological agents in the patients studied here suggests that HBV is not the cause of CNS disease. Although we cannot exclude that HBV contributed to the CNS symptoms experienced by these two patients, determining whether HBV may induce a more severe CNS syndrome, or is simply a passive co-infecting agent, clearly requires additional investigation.

## Ethical approval

Verbal (2003–2006) or written (2006–2011) informed consent was obtained from all recruited patients or close relative. Ethical clearance was granted by the Ethical Review Committee of the Faculty of Medical Sciences, National University of Laos and the Oxford University Tropical Ethics Research Committee, Oxford, UK.

## Funding

JHOP is funded by the Swedish research council FORMAS (grant no: 2015–710). ECH is funded by an ARC Australian Laureate Fellowship (FL170100022). JHOP, VE, XDL are members of the ZIKAlliance Programme of the European Union. This study was supported by the ZIKAlliance project under European Union Horizon 2020 programme (Grant Agreement no: 734548), the European Union's Horizon 2020 research and innovation programme EVAg under grant agreement N° 653316, and Aix-Marseille University. The funders had no role in study design, data collection and analysis, decision to publish, or preparation of the manuscript. The work in Laos was funded by the Wellcome Trust of UK, which also supports Paul Newton, and the Institute of Research for Development (IRD).

## Conflict of interest

The authors declare that they have no conflict of interest.

## Author contributions

Designed the study: JHOP and ADP.

Performed field- and lab-work: JHOP, GP, ADP, PN, MV, MM, SR, VD.

Performed bioinformatics work: JHOP, KA, VE.

Wrote the manuscript: JHOP wrote the initial draft with input from ADP, GP, XdL, PN and ECH. All authors read and approved the manuscript.
